# Effects of Larval Starvation Stress on the Life History and Adult Fitness of Fall Webworm, *Hyphantria Cunea*

**DOI:** 10.3390/insects16040410

**Published:** 2025-04-14

**Authors:** Yuan Zhang, Lin Zhang, Junchao Zheng, Tongpu Li, Lvquan Zhao

**Affiliations:** Collaborative Innovation Center of Sustainable Forestry in Southern China, College of Forestry, Nanjing Forestry University, Nanjing 210037, China

**Keywords:** *Hyphantria cunea*, starvation resistance, complete food deprivation, refeeding, invasive spread

## Abstract

Insects often encounter starvation stress, especially during invasion spread or population outbreaks. The fall webworm, *Hyphantria cunea* (Drury) (Lepidoptera: Arctiidae), is an important invasive pest in China, and the starvation resistance of its larvae determines population spread and resulting outbreak threat. In this study, we investigated the starvation resistance of *H. cunea* larvae and the effects of starvation stress on their life history and adult fitness. Larval starvation resistance increased along with the instar stage, and the second-day sixth instar molt was critical for starvation resistance. The response to starvation stress was reflected in multiple biological indicators observed in *H. cunea*. Complete food deprivation reduced the pupation survival rate of sixth instar larvae, prolonged their developmental duration, reduced pupal and adult body mass, shortened adult forewing length and lifespan, and reduced female egg production. The sixth instar larvae showed a certain ability to recover after refeeding. However, negative impacts of the starvation period on larval life history traits, such as pupation survival rate, pupal and adult body mass, adult longevity, and fecundity, were still observed even after refeeding, and the effects of refeeding were affected by larval starvation duration and refeeding mode. These results suggested that *H. cunea* larvae were highly resistant to starvation, that starvation stress negatively affected their larval life history and adult fitness, and that the pattern of the effects of post-starvation refeeding on larval and adult fitness was related to the duration of starvation and the refeeding mode. The results of this study offer important insights into understanding the physiological response mechanisms of invasive insects under starvation stress.

## Simple Summary

The fall webworm, *Hyphantria cunea* (Drury) (Lepidoptera: Arctiidae), is an important invasive pest in China. During the processes of its invasive spread or population outbreaks, food supplies fluctuate in response to spatial and temporal changes, resulting in frequent starvation stress. In this study, the larval stages were deprived of a food supply and the effects of complete food deprivation and refeeding on larval life history and adult fitness were investigated. *H. cunea* larvae were highly resistant to starvation, but complete food deprivation negatively affected their larval life history and adult fitness. Refeeding after starvation had certain restorative and compensatory effects on the larval life history and adult fitness, with the effect pattern being related to the duration of starvation and refeeding mode. The results are of great significance for understanding the starvation resistance mechanisms of *H. cunea* larvae and for assessing and controlling its spread.

## 1. Introduction

In nature, insect food supplies fluctuate in response to spatial and temporal changes caused by biotic or abiotic factors, which in turn leads to frequent starvation stress, especially during the processes of insect invasion, spread, or population outbreaks [1,2]. As heterotrophic organisms, insects need to sustain their energy supplies for survival through feeding [3]. When the food supply is insufficient or depleted, insects need to rely on the nutrients they have previously acquired or stockpiled to survive and regulate the allocation of their limited resources to make trade-offs between survival and reproduction [4]. Therefore, starvation stress is a physiological challenge for insects [2].

In response to starvation stress, insects adopt different adaptive strategies, such as migration, diapause, or cannibalism [5]. Typically, insects enter a state of slowed growth rate, accompanied by an increase in starvation resistance [6]. While long-term starvation inevitably leads to mass mortality, even short-term starvation can have serious impacts on survival [7,8]. In addition to the depletion of energy reserves, starvation stress leads to a decrease in immunity, with individuals subjected to starvation stress being more susceptible to invasion by harmful microorganisms, such as viruses, than individuals that feed normally, leading to death from multiple stresses [9,10]. For holometamorphic insects, larvae can regulate their growth and development according to their own growth state after experiencing starvation stress. Most insects increase their chances of reacquiring food by prolonging their developmental duration [11]. Some insects can initiate pupation within a short duration after experiencing starvation stress, thereby avoiding starvation stress by entering the pupal stage earlier [12]. Once feeding is resumed, individuals subjected to starvation stress counteract the effects of starvation stress on their growth and development by compensatory feeding or prolonging the developmental duration [13,14,15]. These are considered adaptive mechanisms.

Adult fitness is closely linked to the food supply status during the larval stage [16,17]. Since most lepidopteran insects have a limited ability to replenish nutrients during the adult stage, the energy supply during that stage comes almost entirely from the larval stage’s energy reserve [18]. Therefore, starvation during the larval stage leads to a smaller adult size, shorter lifespan, and reduced reproductive ability, which in turn reduces adult fitness. This phenomenon is known as the “silver spoon effects” [19]. However, following starvation, larvae exhibit partial fitness recovery through ad libitum refeeding, with adults potentially exhibiting high fitness levels following eclosion [20]. Larvae can sense changes in current environmental conditions, and they survive by regulating their growth and development in anticipation of future adverse environments. For instance, insects can redistribute energy resources during metamorphosis, leading to the evolution of body composition, morphology, and other adult characteristics that are more suitable for individual or population development. The most common result is an enhancement of dispersal or flight ability [17,21,22]. Therefore, species differ in the extent to which starvation stress experienced during the larval stage affects adult fitness.

With the increasing growth of international trade and the improvement of transportation networks, the movement of insects around the world has become more frequent [23]. Commodities act as natural hosts for insects and provide “transportation” for insect invasions [24]. Therefore, relative limitations or scarcities in food resources during human-mediated dispersal may lead to insects encountering long-term starvation conditions. In addition, starvation stress is an important factor driving insect dispersal [5]. When there is a large population explosion, insects need to improve their access to food by expanding their feeding range. Insects sometimes have access to sufficient food through a single transfer, and sometimes they need to keep transferring hosts for food, causing them to shift between starvation and feeding states [25]. Therefore, the degree of impact of starvation stress on insect larval life history and adult fitness during both active and passive dispersal is then the key to the successful dispersal of invasive insects and is of great significance in assessing the dispersal risk.

The fall webworm, *Hyphantria cunea* (Drury) (Lepidoptera: Arctiidae), is an important invasive pest in China, which poses a substantial threat to China’s forest resources owing to its wide host range, adaptability, and high reproductive capacity [26,27]. Under natural environmental conditions, *H. cunea* has a limited ability to spread, but it has experienced events, such as population migration and dramatic expansion, within a short time after its invasion of China, and the invaded areas are characterized by trans-regional characteristics [28]. Therefore, we inferred that this process is inevitably accompanied by human assistance. In addition, the old mature larvae of *H. cunea* are voracious feeders. When the quantity or quality of food in the habitat is poor, the larvae need to constantly transfer hosts to feed. Therefore, considering the temporal and spatial uncertainties of food availability during *H. cunea* larvae dispersal, they are likely to face starvation stress. However, the effects of starvation stress suffered by the larval stage on its life history and adult fitness are unknown.

The aim of this study was to clarify the effects of starvation stress on *H. cunea* larval life history and adult fitness during invasion dispersal or population outbreaks. We hypothesized that the life history and adult fitness were affected by starvation stress during the larval stage. We predicted that complete food deprivation negatively affects larval life history and adult fitness, consistent with the “silver spoon effects”. Additionally, we predicted that larval refeeding after starvation alleviates the negative effects of starvation stress on life history and adult fitness. The results of this study offer important insights into understanding the physiological response mechanisms of invasive insects under starvation stress conditions.

## 2. Materials and Methods

### 2.1. Insect Source and Rearing

The larvae of *H. cunea* were collected in August 2023 by removing 30 entire nests from naturally occurring poplar stands (*Populus* × *canadensis* Moench) in rural areas of Lianyungang City, Jiangsu Province (34°36′ N, 119°13′ E). The samples were sent to the laboratory to be reared in plastic containers (17 cm × 11.7 cm × 6.8 cm) with holes at the top for ventilation. They were fed fresh mulberry leaves (*Morus alba*. L.) ad libitum (three times daily supplementation, availability exceeding consumption), and individuals showing abnormal development were removed. Since the larvae of *H. cunea* were highly aggregated during the 1st–4th instar stages, they were reared in groups until the 5th instar larval stage, after which they were reared individually. After pupation, *H. cunea* were transferred to rearing cages containing egg-laying paper for adults to mate and lay eggs on. After the females laid eggs, each egg mass was transferred individually to a plastic container in which moist absorbent cotton was placed in advance to maintain humidity. After hatching, the larvae were reared for one generation for subsequent experiments using the above methods. All the larvae in this experiment were reared in an incubator with a photoperiod of L:D = 16:8 h and a temperature of 25 ± 1 °C.

### 2.2. Starvation Resistance

To clarify the larval starvation resistance, the survival times of larvae across different instars (1st to 6th instar) were compared under complete food deprivation. Ten groups of egg masses were selected, and 15 larvae were chosen from each group on the day of hatching and on the day of 2nd–6th instar larval molting. They were transferred to plastic containers (40 mL) not containing food for starvation, and the larvae were checked for mortality until all the larvae were dead.

### 2.3. Complete Food Deprivation

The results of the above studies indicated that the 6th instar *H. cunea* larvae had strong starvation resistance. The 6th instar larvae accounted for approximately 80% of the feeding amount of the whole larval stage [29]. Therefore, the effects of complete food deprivation on the growth and development, pupation survival rate of 6th instar larval at different day ages, and the fitness of pupae and adults, were investigated. The 6th instar larvae post-molt were fed fresh mulberry leaves ad libitum. Sixth instar larvae entered the wandering stage and stopped feeding on the 5th day after molting. Therefore, the larvae were transferred to containers without food for complete food deprivation treatments on the 1st, 2nd, 3rd, and 4th day after molting.

### 2.4. Refeeding After Starvation

**Figure 1:**
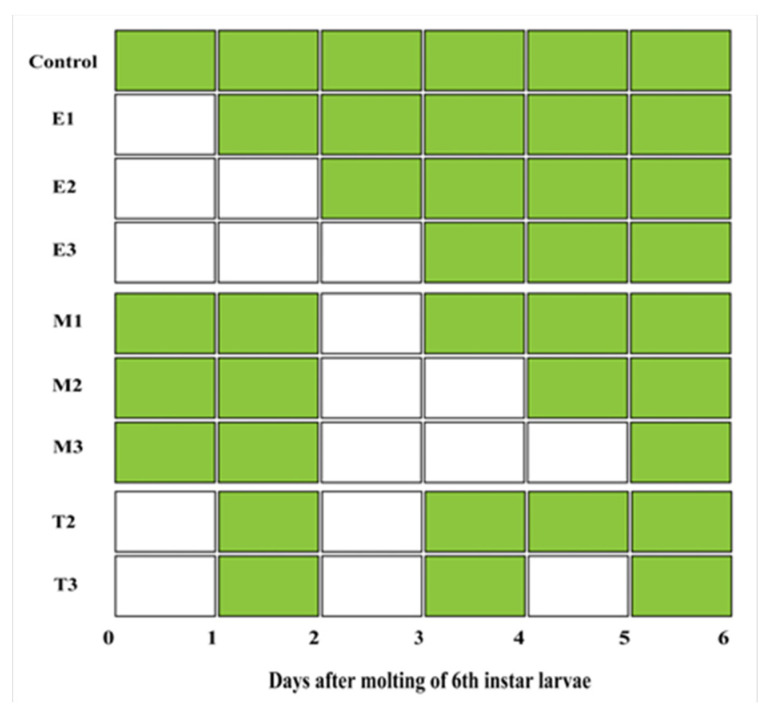
The schedule of feeding (green) and starvation (white) for 6th instar larvae of *H. cunea*. (E1, 2, 3: the 6th instar larvae were starved for 1, 2, and 3 days after molting, and then were fed fresh mulberry leaves ad libitum until pupation. M1, 2, 3: the 6th instar larvae were fed fresh mulberry leaves ad libitum for 2 days after molting, and then they were starved for 1, 2, and 3 days, respectively. Afterwards, the larvae were continuously fed fresh mulberry leaves ad libitum until pupation. T2, 3: the 6th instar larvae were starved for 1 day after molting and refed 1 day. This was repeated two and three times, respectively.

To clarify the recovery ability of larvae to refeed after starvation stress, the 6th instar larvae were divided into three groups. In Group 1 (early starvation refeeding group), the 6th instar larvae were transferred to plastic containers without food on the day of molting. They were starved for 1–3 days and then were fed fresh mulberry leaves ad libitum until pupation. In Group 2 (midterm starvation refeeding group), the larvae were fed fresh mulberry leaves ad libitum for 2 days after molting; they were then transferred to containers and starved for 1–3 days. Afterwards, the larvae were continuously fed fresh mulberry leaves ad libitum until pupation. In Group 3 (intermittent starvation group), the larvae was divided into two ways of treatment. In the first treatment, larvae were starved from the 1st day of molting, provided with fresh mulberry leaves ad libitum on the 2nd day, and then starved again on the 3rd day. Afterwards, they were continuously fed fresh mulberry leaves ad libitum until pupation. In the second treatment, larvae were starved from the 1st day of molting, provided with fresh mulberry leaves ad libitum on the 2nd day, and then starved again on the 3rd day. They were provided ad libitum feeding on the 4th day, deprived of food on the 5th day, and finally maintained on continuous ad libitum feeding from the 6th day until pupation. The details of the experimental design are shown in Figure 1.

### 2.5. Measurement Parameters

To clarify the effects of starvation stress on larval life history and adult fitness, developmental duration and survival rates were measured during the larval stage, pupal body mass was measured during the pupal stage, and adult body mass, longevity, female fecundity, forewing length, and thorax/abdomen ratio were measured during the adult stage. After larvae were subjected to starvation treatments, larval survival and developmental duration were recorded daily. The larvae were considered alive if they moved after being touched on the thorax with a fine bristle brush, and they were considered dead if they did not move. On the 2nd day after pupation, the sex of each larva was identified based on the positions of the 8th and 9th instar germinal pores of the pupa [30]. Each pupa was weighed using an electronic balance (AL104 Mettler-Toledo; Mettler-Toledo Zurich, Switzerland, d = 0.0001 g) and placed in a cage containing egg-laying paper. Adults were weighed on the day they fledged, and the numbers of eggs laid were counted under a microscope. The lifespans of adults were recorded after death, and forewing lengths were measured using a micrometer (0.1 cm). In addition, on the day of adult emergence, the female and male adults were placed in centrifuge tubes, frozen in liquid nitrogen, and stored at −80 °C. Later, they were placed in a drying oven at 70 °C for 24 h. The thorax and abdomen of each adult were separated using a dissecting knife under a microscope, and the dry weights were determined using an electronic analytical balance to calculate their thorax/abdomen ratio.

$$\mathrm{Thorax}/\mathrm{abdomen}\;\mathrm{ratio}\;(\%)=\frac{thorax\;dry\;mass\left(g\right)}{abdomen\;dry\;mass\left(g\right)}$$

### 2.6. Principal Component Analysis (PCA) of Life History Parameters

Variations in life history parameters of H. cunea larvae that were refed after starvation were investigated. A Kaiser–Meyer–Olkin test was employed to assess partial correlations among variables, with a value > 0.5 indicating sampling adequacy. Subsequently, standardized numerical variables were subjected to PCA through the prcomp function. Principal components 1 (PC1) and 2 (PC2) were retained based on their cumulative variance contributions calculated from eigenvalue proportions. Finally, the factoextra package’s fviz_pca_biplot function was utilized to visualize the distribution relationships between samples and variables in the PC1-PC2 space through a biplot representation.

### 2.7. Data Analysis

The experimental data were analyzed using IBM-SPSS v.22.0 (IBM, Armonk, NY, USA). Differences in survival times among larvae of all instar stages were compared using the Kruskal–Wallis test. Survival rate was using the Kaplan–Meier analysis, and the Long-rank test was used to compare larval pupation survival rates. Body masses of pupae and adults, forewing lengths of adults, thorax/abdomen ratios and female fecundity were analyzed using a one-way ANOVA with post hoc Tukey’s multiple range tests. Larval developmental duration, adult lifespans and pupation survival rates with refeeding after larval starvation did not conform to a normal distribution and were analyzed using the Kruskal–Wallis test. In addition, a PCA was performed using R software (version 4.3.0).

## 3. Results

### 3.1. Survival Time

**Figure 2:**
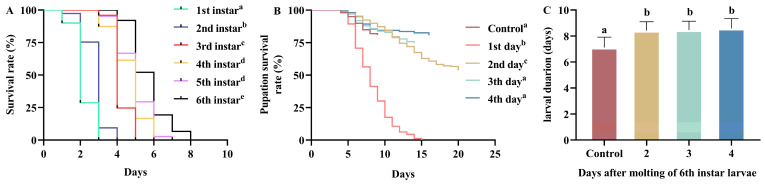
Effect of starvation on the survival and development duration of *H. cunea* larval. (**A**) Survival curves of larvae of different *H. cunea* larval instar stages. (**B**) Pupation survival curves of 6th instar *H. cunea* larvae at different days of molting, under complete food deprivation. Each curve was discontinued once all the larvae in the treatment group had pupated. (**C**) Development duration of 6th instar larvae *H. cunea* larvae at different days. Different letters indicates a significant difference among treatment groups.

Starvation stress significantly affected the survival time of larvae at all instar stages (H = 657.2, df = 5, *p* < 0.001; Figure 2A), and the larval survival time was significantly prolonged as development progressed through each instar stage. The first instar larvae began to die from the first day after hatching, and they were all dead after the third day. However, as the larval instar stage increased, the time until the first death, and the larval survival time were significantly prolonged.

### 3.2. Complete Food Deprivation

Complete food deprivation significantly affected the pupation survival rate of sixth instar larvae at different days of molting (χ^2^ = 854.6, df = 4, *p* < 0.001; Figure 2B). There was a significantly increasing trend in larval pupation survival rate with the delay in starvation treatment. All larvae died when the starvation treatment began on the first day after larval molting. When larvae were starved on the second day of molting, larval pupation survival rate increased to 54%. When larvae were starved starting on the third and fourth day after molting, the pupation survival rate increased to 77% and 79%, respectively, which were significantly higher than the rate when starvation began on the second day after molting (*p* < 0.001) and similar to the pupation survival rate in the non-starvation treatment. In addition, complete food deprivation significantly affected the larval developmental duration (H = 206.9, df = 3, *p* < 0.001; Figure 2C). Complete food deprivation lingered larval developmental duration, but there were no significant differences in developmental duration among the three treatment groups.

**Figure 3:**
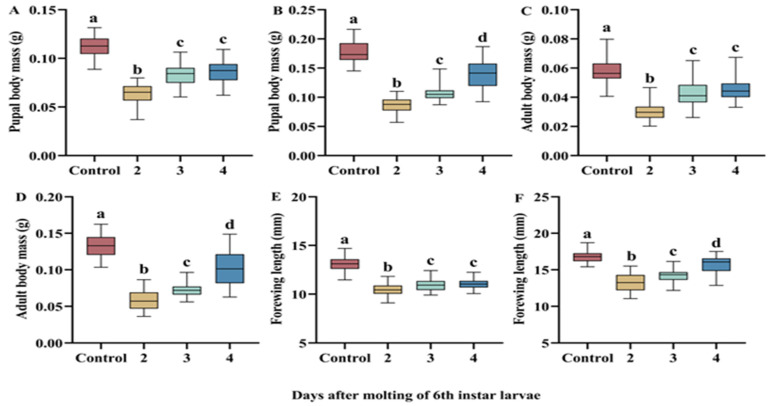
The effect of complete food deprivation on the body size of pupal and adult *H. cunea*. Pupal body mass of male (**A**) and female (**B**). Adult body mass of male (**C**) and female (**D**). Adult forewing length of male (**E**) and female (**F**). Data are shown as means ± SE. The top and bottom of each boxplot represent the upper and lower quartiles, respectively; the horizontal line within the box represents the mean; the whiskers extend to the minimum and maximum values within 1.5 × the interquartile range. Different letters indicate statistically significant differences among groups.

Complete food deprivation in the larval stage had significant effects on pupal body mass (male, F_3,309_ = 316.67, *p* < 0.001; Figure 3A; female, F_3,151_ = 225.05, *p* < 0.001; Figure 3B), adult body mass (male, F_3,298_ = 227.38; *p* < 0.001; Figure 3C; female, F_3,141_ = 219.59, *p* < 0.001; Figure 3D), and forewing length (male, F_3,282_ = 247.51, *p* < 0.001; Figure 3E; female, F_3,138_ = 103.55, *p* < 0.001; Figure 3F). Starvation stress in the larval stage reduced pupal body mass, but with a delay in the beginning of the starvation treatment, pupal body mass tended to increase significantly. The trends of adult body mass and forewing length were consistent with that of pupal body mass.

**Figure 4:**
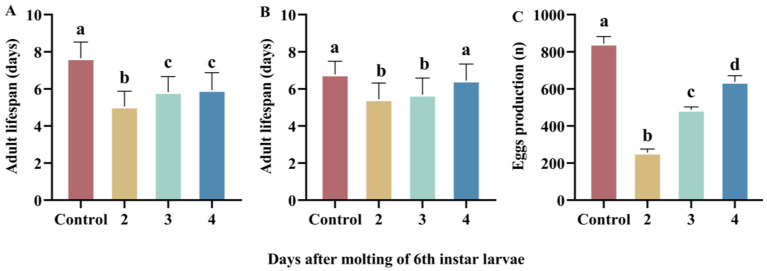
The effect of complete food deprivation on adult fitness of *H. cunea*. Adult lifespans of males (**A**) and females (**B**). Egg production (**C**). Data are shown as means ± SE. Different letters indicate significant difference among treatment groups.

Complete food deprivation in the larval stage significantly affected adult longevity (male, H = 145.15, df = 3, *p* < 0.001; Figure 4A; female, H = 39.61, df = 3, *p* < 0.001; Figure 4B), and female egg production (F_3,82_ = 59.34, *p* < 0.001; Figure 4C). The larval starvation stress shortened the adult lifespan and reduced female egg production, but both traits tended to increase significantly with a delay in the beginning of the larval starvation treatment.

### 3.3. Resilience to Refeeding After Starvation

Modes of refeeding after larval starvation significantly affected the pupation survival rate (H = 51.74, df = 8, *p* < 0.001; Table 1) and developmental duration (H = 166.9, df = 8, *p* < 0.001; Table 1). Compared with the control group, the larval pupation survival rate was not significantly affected by refeeding after intermediate starvation treatment group, whereas it decreased significantly in the intermittent starvation treatment group (*p* < 0.001). Unlike the above two treatment groups, the pupation survival rate in the early starvation treatment group was affected by the duration of starvation. Compared with the control group, refeeding after 1 or 2 days of starvation had no significant effect on the pupation survival rate. However, refeeding after 3 days of starvation significantly decreased the pupation survival rate (*p* < 0.001). In addition, compared with the control group, the larval developmental duration was prolonged with the increased starvation duration in all three treatment groups. Within the same starvation period, the average developmental duration of larvae in the midterm starvation treatment group was significantly shorter than that in the early starvation treatment group (*p* < 0.001).

**Figure 5:**
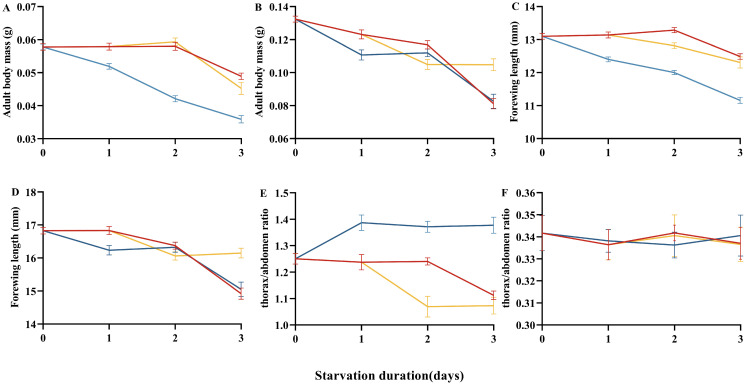
Effects of refeeding mode on the body size of *H. cunea* adults. Adult body mass of males (**A**) and females (**B**). Adult forewing length of males (**C**) and females (**D**). Adult thorax/abdomen ratio of males (**E**) and females (**F**). Data were shown as means ± SE. In all panels, red line represents the 6th instar larvae were starved after molting, and then fed fresh mulberry leaves ad libitum until pupation. Blue line represents the 6th instar larvae that were fed fresh mulberry leaves ad libitum for 2 days after molting and then were starved. Afterwards, the larvae were continuously fed fresh mulberry leaves ad libitum until pupation. Yellow line represents the 6th instar larvae that were starved for 1 day after molting and refed 1 day, with the treatments being repeated.

Modes of refeeding after larval starvation significantly affected pupal body mass (male, F_8,609_ = 112.75, *p* < 0.001; Table 1; female, F_8,264_ = 32.88, *p* < 0.001; Table 1), adult body mass (male, F_8,526_ = 58.55, *p* < 0.001; Figure 5A; female, F_8,261_ = 37.01, *p* < 0.001; Figure 5B), forewing length (male, F_8,483_ = 63.86, *p* < 0.001; Figure 5C; female, F_8,251_ = 21.25, *p* < 0.001; Figure 5D), and thorax/abdomen ratio (male, F_8,298_ = 22.57, *p* < 0.001; Figure 5E; female, F_8,195_ = 0.11, *p* = 0.99; Figure 5F). For male pupae, compared with the control group, the pupal body masses of all three treatment groups decreased significantly with the extended duration of larval starvation, but the average pupal body mass of the early starvation refeeding treatment group was significantly greater than those of the midterm and intermittent starvation treatment groups having the same starvation duration (*p* < 0.001). For female pupae, compared with the control group, the pupal body masses also decreased with the extended duration of larval starvation, and the early and midterm starvation refeeding treatment groups showed similar trends. When larvae resumed feeding after 1 and 2 days of starvation, pupal body masses were similar among the three treatment groups, but when starved for 3 days, the average pupal body mass of the intermittent starvation treatment group was significantly greater than those of the early and midterm starvation followed by refeeding treatment groups (*p* < 0.001).

The trends in adult body mass and forewing length were consistent with those of pupal body mass. However, unlike the changes in pupal body mass, the thorax/abdomen ratio of males in the early starvation refeeding and intermittent starvation treatment groups showed a decreasing trend as the duration of the starvation treatment was prolonged, whereas the thorax/abdomen ratio of males in the midterm starvation refeeding treatment group showed an increasing trend and was significantly larger than those of the males in the early starvation refeeding and intermittent starvation post-feeding treatment groups. The female thorax/abdomen ratios were not significantly affected by the feeding methods, and the female thorax/abdomen ratios of the three treatment groups were maintained at relatively stable levels.

Modes of refeeding after larval starvation significantly affected adult longevity (male, H = 62.29, df = 8, *p* < 0.001; Table 2; female, H = 39.25, df = 8, *p* < 0.001; Table 2) and female egg production (F_8,135_ = 8.55, *p* < 0.001; Table 2). For males, changes in male longevity were similar among the three treatment groups. Compared with the control group, there was no significant change in male longevity after 1 and 2 days of starvation, but when starved for 3 days, male longevity decreased significantly (*p* < 0.001). For females, when starved for 3 days, compared with the control group, only the lifespans of those that resumed feeding after starvation in the early and midterm starvation treatments decreased significantly, and there was no significant change in the lifespans of females in the other treatments. Compared with the control group, the egg production of females in the three treatment groups decreased with the prolonged starvation treatment time. When starved for 3 days, the egg production of females in the early and midterm starvation, followed by refeeding, treatment groups were similar to that of the intermittent starvation treatment group, but they were significantly lower than those in the refeeding treatment groups after 1 and 2 days of starvation (*p* < 0.001).

### 3.4. The Total Biological Response of H. cunea to Starvation Stress

**Figure 6:**
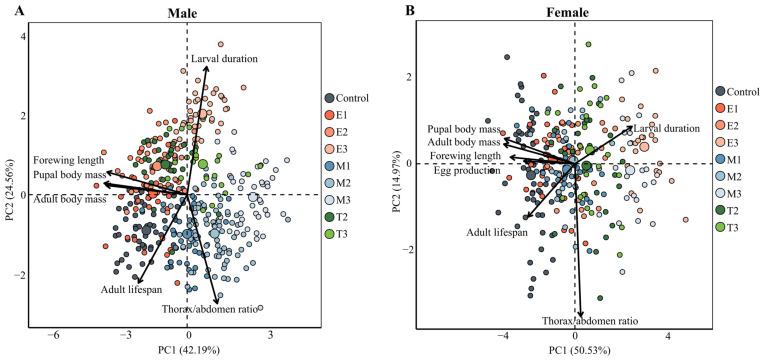
PCA of the life history parameters of *H. cunea* under different refeeding modes after starvation. (**A**) Male; (**B**) Female. Abbreviations are provided in Figure 1.

The life history parameters of *H. cunea* exhibited significant responses to starvation stress, with observed sex-specific differences. For males (Figure 6A), PC1 and PC2 cumulatively accounted for 66.75% of the variation. The adult thorax/abdomen ratio showed negative correlations with larval development duration, pupal and adult body mass, and forewing length. In females (Figure 6B), PC1 and PC2 together explained 65.5% of the variation, with the larval development duration exhibiting negative correlations with pupal and adult body mass, lifespan, egg production and thorax/abdomen ratio. Additionally, positive correlations were observed between pupal and adult body mass, forewing length, egg production, and lifespan when comparing male and female individuals.

## 4. Discussion

Food plays a critical role in the life history of insects as a major energy source for growth and development [5]. In this study, we modeled how *H. cunea* larvae may encounter starvation stress during dispersal by depriving the larval stages of a food supply. The response to starvation stress was reflected in multiple biological indicators observed in *H. cunea*. Consistent with our expectations, when larvae were subjected to complete food deprivation, they displayed reduced pupation survival rate, prolonged developmental durations, decreased pupal and adult body masses, shortened adult forewing lengths and lifespans, and reduced female fecundity levels. Thus, complete food deprivation negatively affected larval life history and adult fitness. However, counter to our expectations, larvae that underwent refeeding after starvation had weaker recovery levels for characteristics defining life history and adult fitness, and the patterns of the starvation stress effects were related to the duration of starvation and the modes of refeeding.

Starvation resistance in insects is influenced by factors such as age, sex, and environmental conditions [31,32]. Our results suggested that the starvation resistance of larvae increased along with the developmental instar stage. Similarly, the third instar larvae of *Dendrolimus pini* showed greater starvation resistance than the first instar larvae [8]. This phenomenon may be related to the energy reserve levels of the larvae, because larval body mass increases during development and the energy reserves of insects are positively correlated with body mass [33]. Therefore, more developed instar larvae have more energy reserves than less developed instar larvae, and thus, they show greater resistance to starvation. In addition, the rapid increase in pupation survival rate with the delay of starvation treatment further validated our speculation. All the larvae died when the starvation treatment was initiated on the first day of the sixth larval molt, whereas the pupation survival rate increased significantly when the starvation treatment was initiated from the second day onwards. This suggested that the second-day sixth instar molt was critical for starvation resistance. Similar results have been shown in studies of other holometamorphic insects [34,35]. The above results indicated that starvation stress significantly and negatively affected the survival of *H. cunea* larvae and that the effect was influenced by the developmental instar stage and age of the larvae.

The developmental duration of insects is highly plastic and is strongly related to the quality and quantity of nutrients acquired [36]. Here, complete food deprivation prolonged the duration of larval development. This suggested that larvae prolonged their developmental duration to increase their chances of refeeding when subjected to starvation stress. In fact, prolonging the duration of larval development would increase the risk of predation or infection by pathogenic pests. Therefore, the opportunity to refeed may decrease their chances of survival [37]. The duration of larval development was not further prolonged with the delay in the starvation treatment. This may be because larval development was influenced by a critical threshold for pupation to ensure that it did not unduly delay maturation in harsh environments [11]. However, in contrast to the above results, for *Osmia lignaria*, starvation resulted in an early entry into the pupal stage and a significant shortening of the larval developmental duration [12]. These results suggested that the developmental duration was plastic when larvae were subjected to starvation stress and that phenotypic expression patterns differed among species.

Starvation not only affects larval growth and development, but it also influences the life history traits of their subsequent developmental stages [38]. For example, starvation in the larval stage reduces pupal body mass and female fecundity in *Pieris brassicae* [39], whereas in *Lycaena tityrus*, starvation in the larval stage not only results in reduced adult body mass and forewing length, but it also reduces adult flight performance [40]. Our results are consistent with the findings that complete food deprivation during the larval stage not only reduced pupal and adult body masses, but it also shortened both adult forewing length and lifespan and reduced female fecundity. The degree of energy reserves in the larval stage determined the fitness of pupae and adults due to the degradation of the adult *H. cunea*’s mouthparts, which resulted in the inability of adults to take food to replenish nutrients after they have fledged [18]. When the larval stage experienced complete food deprivation, its energy intake was reduced, which in turn led to a decrease in the body mass of the pupal or adult. In general, adult fitness is related to body size, and larger individuals are more dominant in dispersal, mate finding, mating, and reproduction [41,42]. Therefore, starvation stress suffered during the larval stage resulted in a smaller adult size, which in turn led to decreased fecundity and a shorter lifespan, thus decreasing adult fitness. These results suggested that complete food deprivation during the larval stage negatively affected the fitness of pupae and adults, which further confirmed our prediction that the “silver spoon effects” would be fulfilled.

In general, insect refeeding after experiencing starvation stress restores its growth and development, thereby mitigating the effects of starvation stress. For example, for *Sitbion avenae*, larval survival decreases with increasing starvation duration when they refeed after 1–4 days of starvation, but adult reproductive capacity and longevity are not affected [43]. Our study results were similar, with adult longevity and fecundity not being significantly affected by the resumption of feeding after 1–2 days of starvation during the larval stage. This may be because when insects refeed after starvation, they accumulate certain key substances and metabolites [44,45]. For example, refeeding after the starvation of *Drosophila* larvae results in higher adult fat reserves [46], whereas *Apis mellifera* larvae refeeding after starvation shows increases in adult glycogen reserves and juvenile preserving hormone titers [47]. Increases in these substances are beneficial in maintaining their adult fitness. However, except individuals that resumed feeding after 1 day of starvation, among the early sixth instar larvae that fully recovered, most were unable to compensate for the body mass loss caused by starvation stress. Thus, they were reduced in size and showed marked decreases in survival and recovery as starvation duration increased. Similar trends have been observed in *P. brassicae* and *D. pini* [15,39], suggesting that starvation stress is destructive [13]. The above results suggested that *H. cunea* larvae had weak abilities to recover from refeeding after starvation stress and that the effects of refeeding were regulated by the duration of starvation.

Certain lepidopteran larvae accelerate pupation by reducing body size under starvation and prolong the feeding period after refeeding to enhance nutrient accumulation, ultimately restoring their original body dimensions [8,12]. In contrast, when *H. cunea* larvae were refed after starvation, their developmental duration was significantly prolonged and their body mass decreased. Similar findings have been documented in *Orgyia antiqua* [48]. Two potential explanations exist for these phenomena. The first explanation hypothesizes that when larvae experience starvation stress, the decline in juvenile hormone concentration accelerates their pupation, thereby leading to a reduction in pupal mass. However, upon refeeding, the juvenile hormone concentration rapidly rebounds, which delays the pupation timing while allowing partial recovery of pupal mass [49,50]. The second explanation hypothesizes that although larvae exhibit prolonged developmental duration after starvation and refeeding, the nutrients acquired during this extended period are not fully allocated to mass recovery [22]. For instance, in *Manduca sexta*, when larvae refeeding after starvation, their gut may prioritize the absorbed nutrients for repairing damaged tissues or supporting immune functions rather than transferring them to the fat body for energy storage [51,52].

Our results suggested that there were differences in the recovery abilities of larvae under different refeeding methods. For males, individuals that were refed after early starvation recovered their body masses better than those that were refed after midterm starvation for the same starvation duration. Most larvae in the middle of the sixth instar had the ability to pupate (Figure 2B). Therefore, we speculated that the larvae could choose to sacrifice their body size to survive when they were subjected to starvation stress. In contrast, early-starved larvae only fed to maintain survival, as well as growth and development, and to mitigate the effects of starvation stress by further extending the larval developmental duration. For females, intermittently starved individuals recovered their body masses better than those who were refed after early starvation and midterm starvation as the duration of starvation treatment increased. This suggested that females may have developed a buffering mechanism against starvation stress after encountering intermittent starvation, and it was evident that this adaptation was beneficial to adult fitness, but this buffering mechanism needs to be further investigated.

The thorax/abdomen ratio of adults is related to their energy allocation and is regulated by environmental conditions during the larval stage [53,54]. In general, insects reduce their input to the thorax and allocate more energy to the abdomen after encountering starvation stress to ensure female fecundity [55]. However, for *Bicyclus anynana*, larval starvation stress leads to an increase in the adult thorax ratio, which improves adult flight dispersal ability [53]. Our results showed that starvation stress altered the male thorax/abdomen ratio, whereas the female thorax/abdomen ratio was not affected. This phenomenon may be related to the gender differences in trade-off strategies between flight and reproduction. For adult *H. cunea*, males need to search for mates by flight after fledging. Therefore, for males that resumed feeding after starvation in the middle of the sixth instar stage, the increase in the thorax/abdomen ratio indicated that relatively more nutrients were allocated to the thorax. This contributed to the increased flight dispersal ability and, consequently, increased their chances of obtaining a mate, which is favorable for adult fitness. In females, reduced flight demands lead to the maintenance of a stable abdominal investment ratio, through which reproductive capacity is ensured and long-term population viability is promoted. The above results indicated that there was adult fitness trait compensation when the larvae resumed feeding after starvation.

In addition, our results provide new insights into the control and management of *H. cunea*. For example, when a population outbreak occurs, monitoring should be strengthened, and control measures should be flexibly adjusted based on the developmental progress of the insects under natural environmental conditions. In areas having a high population density, the artificial removal of host plant leaves and nets, or the spraying of botanical insect antifeedants on the host plants, should be employed to inhibit *H. cunea* growth or induce mortality, thereby suppressing their population density. For invasive insects, the successful dispersal of single or breeding individuals leads to population outbreaks and the colonization of invaded sites [56]. Therefore, during the human-mediated population spread process, the management of goods transportation can be optimized. For example, reducing the food supply for insects during transport and evaluating the insects’ developmental status upon arrival at the destination. Measures, such as isolation and delayed treatment, can be implemented to extend the period of starvation stress on the larvae, thereby reducing the survival rate of the invasive population, thereby blocking or delaying the spread and diffusion of the population.

## 5. Conclusions

In summary, *H. cunea* larvae showed a certain degree of starvation resistance, and sixth instar larvae were highly resistant to starvation, with some larvae surviving complete food deprivation and completing the remainder of their life history. However, complete food deprivation during the larval stage had significant negative impacts on their life history and adult fitness, and their ability to recover from refeeding after experiencing starvation stress was weak. Therefore, our findings provide critical guidance for studying the mechanisms underlying starvation resistance in *H. cunea* larvae. Based on the current findings, we suggest that future studies explore the molecular regulatory mechanisms of *H. cunea* larvae to gain a more comprehensive understanding of their starvation resistance strategies.

## Figures and Tables

**Table 1:** Effects of refeeding mode on the pupation survival rate, developmental duration of 6th instar larvae, and pupal body mass of *H. cunea*.

Treatment	Pupation Survival Rate (%)	Larval Duration (Days ± SE)	Pupal Body Mass (mg ± SE)	
			Male	Female
Control	82.7 a	7.1 ± 0.1 a	111.77 ± 1.08 a	177.63 ± 2.57 a
E1	82.0 a	8.6 ± 0.1 b	107.55 ± 1.13 a	164.19 ± 2.07 b
E2	78.0 ab	10.7 ± 0.1 cf	107.19 ± 1.20 a	157.30 ± 2.98 b
E3	61.3 bc	12.7 ± 0.1 d	94.52 ± 1.07 c	118.05 ± 4.17 c
M1	84.7 a	7.8 ± 0.1 e	94.27 ± 1.07 c	153.45 ± 3.27 b
M2	78.7 ab	8.2 ± 0.2 be	82.46 ± 1.26 e	150.28 ± 2.78 b
M3	74.7 ab	9.7 ± 0.2 f	70.90 ± 1.29 f	119.27 ± 4.64 c
T2	62.0 bc	9.5 ± 0.1 f	100.85 ± 1.58 b	150.86 ± 3.37 b
T3	54.0 c	10.2 ± 0.1 f	87.71 ± 2.22 cd	153.02 ± 4.29 b

Abbreviations are provided in Figure 1. Different letters in the same column indicate significant differences between treatment groups.

**Table 2:** Effects of refeeding mode on the adult longevity and female fecundity of *H. cunea*.

Treatment	Adult Lifespan (Days ± SE)		Egg Production (n ± SE)
	Male	Female	
Control	7.7 ± 0.2 a	6.8 ± 0.1 a	840.7 ± 36.2 a
E1	7.5 ± 0.2 a	6.5 ± 0.2 a	805.9 ± 40.1 ab
E2	7.5 ± 0.1 a	6.4 ± 0.2 ab	800.8 ± 39.1 ab
E3	6.3 ± 0.1 b	5.5 ± 0.2 b	560.3 ± 30.0 c
M1	7.4 ± 0.2 a	6.2 ± 0.2 ab	796.0 ± 43.2 ab
M2	7.4 ± 0.1 a	6.3 ± 0.2 ab	777.6 ± 42.4 ab
M3	6.3 ± 0.2 b	5.5 ± 0.2 b	541.0 ± 37.0 c
T2	7.5 ± 0.2 a	6.1 ± 0.2 ab	656.1 ± 27.7 bc
T3	6.7 ± 0.5 b	5.9 ± 0.2 ab	633.1 ± 31.2 bc

Abbreviations are provided in Figure 1. Different letters in the same column indicate significant differences among treatment groups.

## Data Availability

The data presented in this study are available on request from the corresponding author.
